# Effect of SiC Nanowhisker on the Microstructure and Mechanical Properties of WC-Ni Cemented Carbide Prepared by Spark Plasma Sintering

**DOI:** 10.1155/2014/673276

**Published:** 2014-06-05

**Authors:** Xiaoyong Ren, Zhijian Peng, Zhiqiang Fu, Chengbiao Wang

**Affiliations:** Key Laboratory on Deep GeoDrilling Technology of the Ministry of Land and Resources, School of Engineering and Technology, China University of Geosciences, Beijing 100083, China

## Abstract

Ultrafine tungsten carbide-nickel (WC-Ni) cemented carbides with varied fractions of silicon carbide (SiC) nanowhisker (0–3.75 wt.%) were fabricated by spark plasma sintering at 1350°C under a uniaxial pressure of 50 MPa with the assistance of vanadium carbide (VC) and tantalum carbide (TaC) as WC grain growth inhibitors. The effects of SiC nanowhisker on the microstructure and mechanical properties of the as-prepared WC-Ni cemented carbides were investigated. X-ray diffraction analysis revealed that during spark plasma sintering (SPS) Ni may react with the applied SiC nanowhisker, forming Ni_2_Si and graphite. Scanning electron microscopy examination indicated that, with the addition of SiC nanowhisker, the average WC grain size decreased from 400 to 350 nm. However, with the additional fractions of SiC nanowhisker, more and more Si-rich aggregates appeared. With the increase in the added fraction of SiC nanowhisker, the Vickers hardness of the samples initially increased and then decreased, reaching its maximum of about 24.9 GPa when 0.75 wt.% SiC nanowhisker was added. However, the flexural strength of the sample gradually decreased with increasing addition fraction of SiC nanowhisker.

## 1. Introduction

WC-based cemented carbide is a class of important tool material, which has been applied in metal cutting, wood machining, rock drilling, and electronics industries because of its high hardness, good wear resistance, and excellent toughness [1–3]. Now cemented carbides are also increasingly used as seal rings, linings, valves, jet nozzles, saw blades, fluid mixers, and conveyor belt scrapers due to their excellent wear resistance and chemical stability [4, 5]. Generally, cobalt (Co) is used as a binder metal in cemented carbides because of its excellent wetting ability to WC and the outstanding mechanical strength of WC-Co [6]. However, the relative low corrosion resistance of WC-Co and high price of Co have limited the applications of cemented carbides as wear parts to some extent in corrosive/oxidative environments, where the components may be expected to remain in service for several years. Moreover, the leaching of Co-based binder can also be hazardous for health since Co is carcinogenic [5]. Thereby, considerable efforts have been focused on the partial or complete replacement of Co by other metallic binders in recent years. Now, Ni is regarded as a desirable one since it presents similar wetting ability to WC, and these cemented carbides exhibited higher corrosion and oxidation resistance than their WC-Co counterparts as well [7–10]. However, because Ni has much lower hardness and strength values than Co, correspondingly WC-Ni cemented carbides also possess relatively lower values than WC-Co cemented carbides, which limits the complete substitution of WC-Ni with WC-Co in applications [8, 11–13].

According to Hall-Petch relation, the hardness of cemented carbides can be improved with the decrease in WC grain size [3, 12–17]. Therefore, the development of finer grained WC-Ni becomes important to enhance the hardness of the WC-Ni cemented carbides. In order to suppress the grain growth during sintering, fast sintering techniques, such as spark plasma sintering (SPS) and high frequency induction heating sintering (HFIHS), and grain growth inhibitors, such as VC, TaC, and Cr_3_C_2_, are always used [12, 13, 17–20]. Rong et al. [12] reported that ultrafine WC-Ni cemented carbides with average WC grain size of about 330 nm could be fabricated by SPS with VC and TaC as grain growth inhibitors at 1350°C. The hardness of WC-6 wt.% Ni was about 24 GPa, which was even higher than that of the WC-6 wt.% Co cemented carbides reported in [14]. In [15], the WC-10Ni cemented carbides with grain size of about 490 nm have been fabricated using HFIHS at about 1250°C under 60 MPa pressure. The hardness of the prepared WC-10Ni (about 1750 HV) was similar to that of the WC-10Co (about 1775 HV) prepared under the same condition in that work.

The hardness and flexural strength of cemented carbides can also be improved by adding some materials with higher hardness or toughness into them [11, 21, 22]. Correa et al. reported that the hardness and strength of WC-Ni cemented carbides could be improved by adding SiC powder into the composite system [11]. The Vickers hardness of WC-10Ni-Si was similar to that of the conventional WC-10Co. Ultra-hard material cBN was added to WC-Ni cemented carbides in [21]. The results indicated that the Vickers hardness of WC-Ni-cBN cemented carbides increased from 2100 to 3200 HV with the added fraction of cBN increasing from 0 up to 50 vol.%, but the flexural strength decreased from 1950 to 1250 MPa [21].

Because SiC whiskers have high strength, high elastic modulus, and good thermal stability, many studies have focused on the composites reinforced by them [22–25]. In [22], WC-Ni cemented carbides with small amount of SiC whisker (no more than 0.87 wt.%) were fabricated by hot-press sintering (HPS) at 1400°C. The flexural strength of WC-10Ni cemented carbides increased from about 1300 to 1700 MPa with 0.53 wt.% SiC whisker. Wu et al. [23] have studied the effect of SiC whisker addition on the mechanical properties of Ti(C,N)-based cermets prepared by vacuum sintering [23]. The results indicated that the fracture toughness could be improved about 29% after 1.0 wt.% SiC whisker was added compared with the cermets without whisker. The toughening mechanisms of the SiC whisker were characterized as crack deflection, whisker bridging, and whisker pulling out. However, Peng et al. [25] reported that due to the smaller scale and higher specific surface area of SiC nanowhisker, the SiC nanowhiskers were hard to uniformly dispersed when the content was higher than 2.5 wt.% and the SiC nanowhiskers would react with Ni to form Ni_2_Si phase during SPSed TiCN-based cermets.

Because of the rapid heating rate and unique joule heating principle of SPS, it was expected that the added SiC nanowhisker might be kept in its original state in the SPSed WC-Ni cemented carbides while the growth of WC grains was inhibited, hopefully improving the mechanical properties of the materials, especially the mechanical strength due to the toughening effect of the added high strength whisker, which was difficult for the HPSed WC-Ni with SiC whisker [22]. Therefore, in this paper, WC-8 wt.% Ni cemented carbides with varied fractions (0–3.75 wt.%) of SiC nanowhisker were fabricated at 1350°C by SPS with the assistance of VC and TaC as WC grain growth inhibitors on the basis of our previous work on WC cemented carbides with different amounts of Ni (6–10 wt.%) as presented in [12]. The effects of SiC nanowhisker addition on the phase composition, microstructure, and mechanical properties of the prepared WC-Ni cemented carbides were investigated.

## 2. Experimental Procedure

### 2.1. Sample Preparation

**Figure 1:**
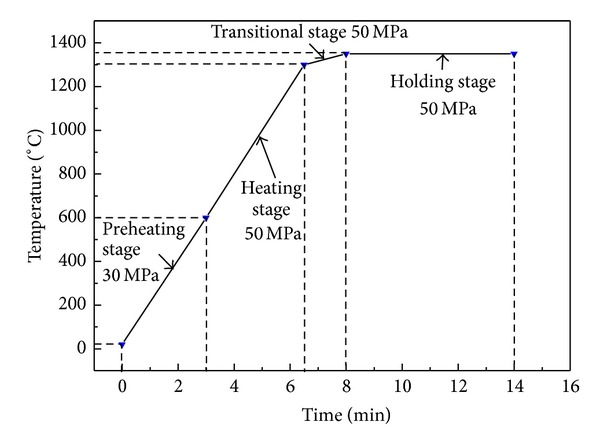
Schematic representation of the temperature and pressure varied with the heating time during SPS.

The applied tungsten carbide (WC), hydroxyl-nickel (Ni), vanadium carbide (VC) and tantalum carbide (TaC) powders, and silicon carbide nanowhisker in this work were all commercially available. Their purity, oxygen content, and size are listed in Table 1. The nominal compositions of the designed samples are presented in Table 2. During processing, the raw powders were deliberately mixed together at different milling stages and milled for disparate times in a high energy attrition mill (model: SY-1, China) with YG-6 cemented carbide balls (ISO: K20) as grinding ball and absolute alcohol as milling medium. In the first stage, the mixture of WC, VC, and TaC powders was milled for 44 h at a speed of 300 rpm, and in the second stage, Ni powder and SiC nanowhisker were added into the mixture and milled for further 4 h at a relatively low speed of 80 rpm, to avoid the deformation and agglomeration of Ni particles and the breakage of SiC whiskers during attritional milling. For milling, the mass ratio of ball to powder was 10 : 1 and that of powder to absolute alcohol was 3 : 1. After milling, the powder slurries were dried in a vacuum oven at 35°C under a pressure of 0.01 MPa. Then, the dried powders were crashed and sieved into fine powders. After that, the prepared composite powder was loaded in a cylindrical graphite die with an inner diameter of 20 mm and an outer diameter of 50 mm and sintered by SPS (Model: SPS-1050T, Japan) in vacuum (≤6 Pa). The sintering process was divided into four stages: preheating, heating, transitional, and holding stages, as shown in Figure 1. In the preheating stage, the temperature was increased from room temperature to 600°C in 3 min with an axial pressure of 30 MPa. After this stage, the axial pressure was increased to 50 MPa and the temperature increased from 600 to 1300°C in 3.5 min. There was a transitional stage of 1.5 min between the heating and holding stage at the temperatures from 1300 to 1350°C to ensure the temperature smoothly into the holding stage. In the holding stage, the temperature and axial pressure were kept 1350°C and 50 MPa for 6 min. After sintering, the finally obtained specimens were disks of approximately 20 mm in diameter and 5 mm in height.

### 2.2. Materials Characterization

Before characterization, all the samples were firstly ground, machined, and mirror-polished into rectangular bars with a dimension of about 2 mm × 3 mm × 15 mm. The flexural strength was measured at room temperature by three-point bending method with an AG-IC 20 kN Shimazu tester. During the testing, the loading rate was 0.5 mm/min and the span length was 10 mm. The bulk Vickers hardness (GPa) was evaluated by a LECO DM-400 hardness tester with an indenting load of 1 kgf and a dwell time of 20 s. For each sample, the values of flexural strength and Vickers hardness are the average of five independent measurements.

In addition, the phase compositions of the as-prepared samples were identified by X-ray diffraction (XRD, D/max2550HB+/PC, Cu K*α* and *λ* = 1.5418 Å) through continuous scanning mode with a speed of 5 °/min. The microstructure of the specimens was examined by scanning electron microscope (SEM, LEO 1530) on the fractured and etched surfaces. Elemental analysis of the compositional phases was performed by an energy dispersive X-ray (EDX) spectroscope attached to the SEM. In addition, the etching was carried out on polished samples in a Murakami's reagent (1 g potassium ferricyanide, 2 g potassium hydroxide, and 30 g water) for about 2 min at room temperature. The binder mean free path (*λ*) and carbide grain size (*d*_WC_) of the sample were calculated by the linear intercept method from the etched surface SEM images [26].

## 3. Results and Discussion

### 3.1. Phase Compositional Analysis

**Figure 2:**
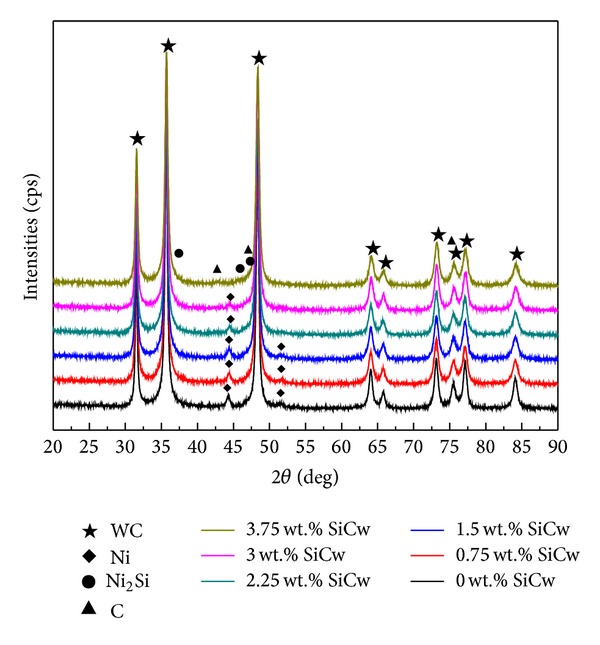
XRD patterns of the as-prepared WC-Ni cemented carbides with varied fractions of SiC nanowhisker.

Figure 2 shows the XRD patterns of the as-prepared WC-Ni cemented carbides with various fractions of SiC nanowhisker. It can be seen that when the added fraction of SiC nanowhisker was lower than 3 wt.%, only WC hard phase (JCPDS card: 51-0939) and Ni binder metal phase (JCPDS card: 65-0380) were identified. However, in the sample with 3.75 wt.% SiC nanowhisker, two series of new phases, Ni_2_Si (JCPDS card: 03-1069) and graphite (JCPDS card: 50-1082), were detected. Moreover, with increasing fraction of SiC nanowhisker, the peak intensity of the Ni phase gradually faded out and even was hard to be detected in the sample with 3.75 wt.% SiC nanowhisker. Ni_2_Si phase was also detected during SPSed TiCN-based cermets in [25]. Jackson et al. [27] and Schiepers et al. [28] all reported that SiC ceramics would react with Ni in vacuum when the temperature was in the range of 700 to 1150°C to form Ni_2_Si and graphite. But the reaction process was diffusion controlled and the reaction rate was very slow. The expedite reaction between the Ni and SiC nanowhisker in this work and [25] may be attributed to the unique plasma sintering model of SPS and the high special surface energy of SiC nanowhisker. The reaction also caused the reduction of the amount of Ni in the sample, resulting in weakening intensity of the Ni phase. In addition, due to the limit of XRD detection, the peak of SiC nanowhisker was not found in the XRD patterns.

### 3.2. Microstructural Characterization

**Figure 3:**
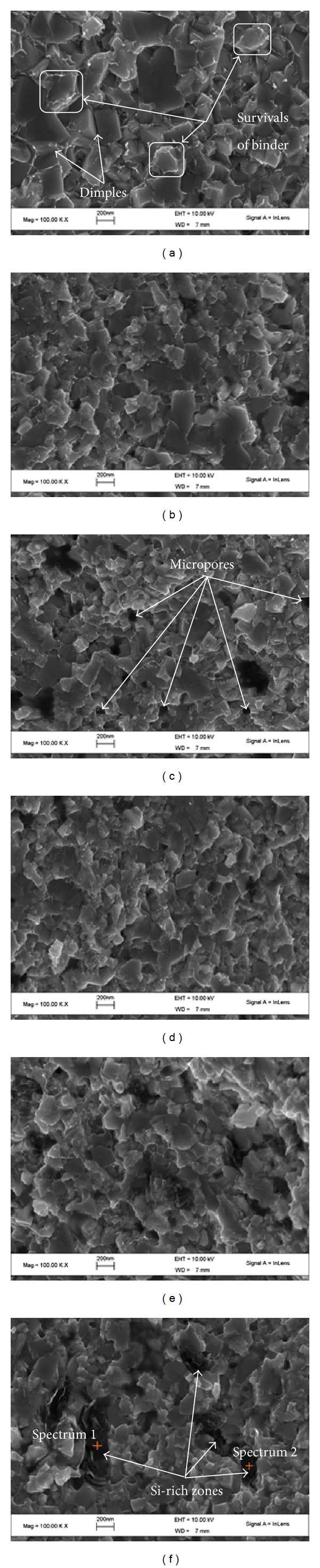
Typical SEM micrographs on the fractured surfaces of the prepared WC-Ni cemented carbides with varied fractions of SiC nanowhisker: (a) 0 wt.%, (b) 0.75 wt.%, (c) 1.5 wt.%, (d) 2.25 wt.%, (e) 3.0 wt.%, and (f) 3.75 wt.%.

Figure 3 displays typical SEM micrographs on the fractured surfaces of the prepared WC-Ni cemented carbides with various fractions of SiC nanowhisker. It can be seen that in the sample without SiC nanowhisker (as shown in Figure 3(a)), most of the fracture was through the Ni binder phase, characterized by the deep dimples and survival of binder phase, demonstrating that there was a relatively strong combination between WC and Ni binder.

However, with increasing fraction of SiC nanowhisker in the samples, the survival of Ni binder on the WC grains was reduced and many faceted planes appeared on the fractured surface. Compared with the sample without SiC nanowhisker, the samples added with SiC nanowhisker possess more micropores as observed on their fractured surface (as shown in Figure 3(c)), which was consistent with the result of their relative density. The reduction of the binder survival and the increased amount of micropores could be all caused by the reaction between Ni and SiC nanowhisker, suppressing the migration of binder phase during liquid sintering. Moreover, with increasing fraction of SiC nanowhisker, more and more aggregates (black in the micrographs as shown in Figure 3(f)) can be observed on the fractured surface. In order to figure out the composition of the black aggregates, EDX spectra were recorded on the aggregates as marked on the micrograph in Figure 3(f). The calculated results from EDX spectra are listed in Table 3. The results revealed that the black aggregates were Si-rich zones. Considering the compositions of the prepared initial composite powders, it could be suggested that the black aggregates may be formed by the agglomeration of the SiC nanowhisker. It should be explained that the high content of oxygen atoms on the two recorded places may be resulted from the oxidation or contamination of the sample surface.

**Figure 4:**
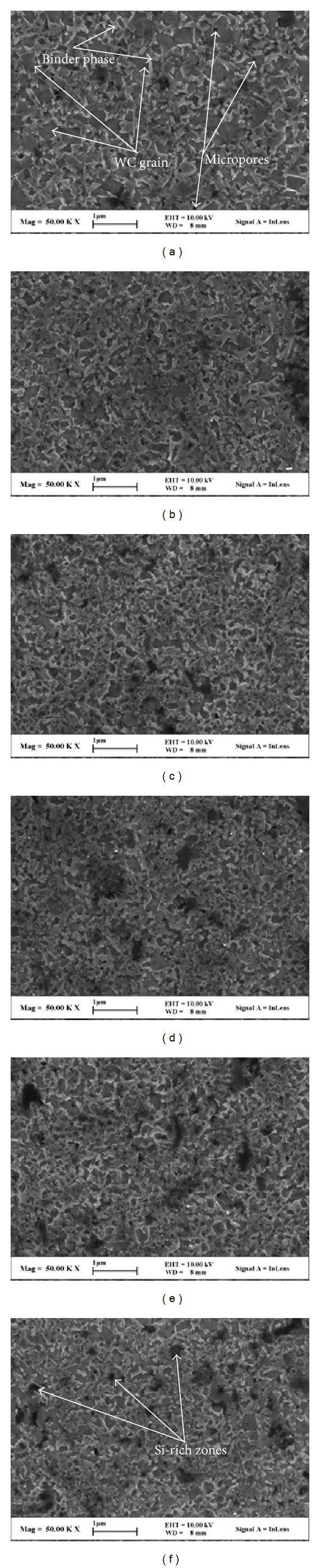
Typical SEM micrographs on the etched surfaces of the prepared WC-Ni cemented carbides with varied fractions of SiC nanowhisker: (a) 0 wt.%, (b) 0.75 wt.%, (c) 1.5 wt.%, (d) 2.25 wt.%, (e) 3.0 wt.%, and (f) 3.75 wt.% (etched by Murakami's reagent for 2 min).

Figure 4 presents typical SEM micrographs on the etched surfaces of the prepared WC-Ni cemented carbide with varied fractions of SiC nanowhisker. It can be calculated by the linear intercept method that the average WC grain size *d*_WC_ was about 400 nm and the average mean free path of binder *λ* was about 20 nm for the sample without SiC nanowhisker, demonstrating that ultrafine WC-Ni cemented carbide was fabricated at 1350°C by SPS with the assistance of VC and TaC as grain growth inhibitors. After the addition of SiC nanowhisker into the samples, the average WC grain size decreased to about 350 nm as shown in Figures 4(b)–4(f). As mentioned in Section 3.1, the reaction between Ni and SiC nanowhisker would reduce the active sites of Ni during the SPS. And the formation of Ni_2_Si and graphite would decrease the wetting ability of the binder phase and suppress the solution of WC in the binder phase. Therefore, the WC grain growth caused by the solution/reprecipitation of WC would be suppressed. On the other hand, the SiC nanowhisker may also suppress the migration of WC before the formation of liquid phase. Moreover, the decrease in the wetting ability of the binder phase also caused the uneven distribution of the binder phase and more micropores as shown in Figures 3(c) and 4(c). With increasing fraction of SiC nanowhisker, more and more Si rich black aggregates were observed on the surface of the samples, resulting in the increase in defects in the sample, which would decrease the hardness and flexural strength of the sample.

### 3.3. Mechanical Properties

**Figure 5:**
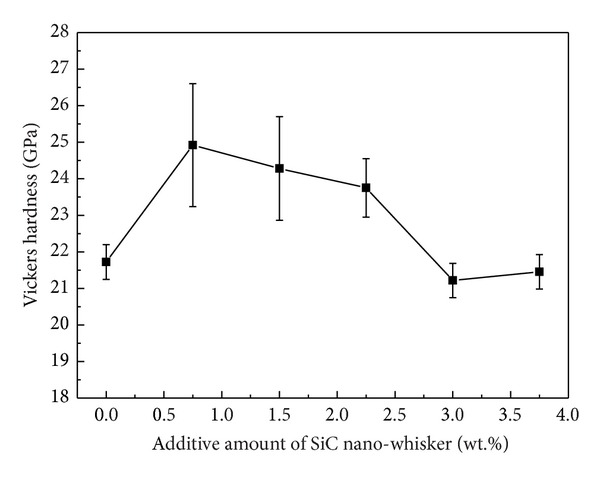
The Vickers hardness of the as-prepared WC-Ni cemented carbides with varied fractions of SiC nanowhisker.

In order to understand the addition effect of SiC nanowhisker on the Vickers hardness of WC-Ni cemented carbides, the Vickers hardness is drawn as a function of the added fraction of SiC nano-whisker in Figure 5. From this figure, it can be seen that with increasing fraction of SiC nanowhisker, the Vickers hardness of the samples initially increased and then decreased. When the added fraction of SiC nanowhisker was 0.75 wt.%, the hardness of the samples reached its maximum of about 24.9 GPa, which was even higher than the hardness of ultrafine WC-6Ni cemented carbides (about 24 GPa) obtained in [12]. The increase in Vickers hardness could be attributed to the reduced average size of WC grains when appropriate amount of SiC nanowhisker was added. However, with increasing fraction of SiC nanowhisker, the micropores and defects increased in the sample due to the existence of Ni_2_Si and graphite produced by the reaction between the Ni and SiC nanowhisker, which was harmful to the sample hardness. Therefore, when the added fraction of SiC nanowhisker was higher than 0.75 wt.%, the hardness of the sample gradually decreased. What is more, when the added fraction of SiC nanowhisker was higher than 2.25 wt.%, the Si-rich aggregates became more and more serious as observed in Figures 3(f) and 4(f), which would also decrease the hardness of the cemented carbides.

**Figure 6:**
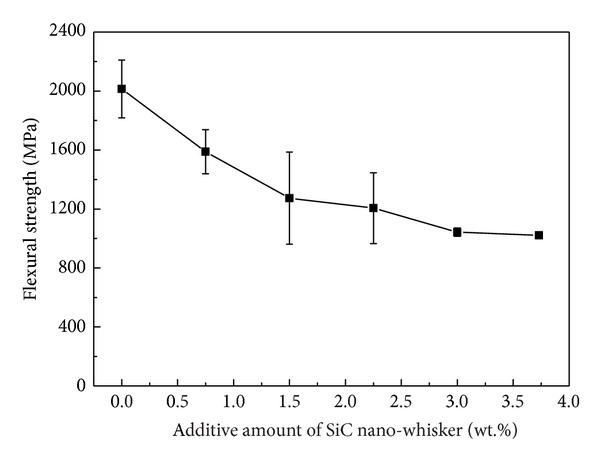
The flexural strength of the as-prepared WC-Ni cemented carbides with varied fractions of SiC nanowhisker.

Figure 6 illustrates the variation of the flexural strength of the obtained WC-Ni cemented carbides with the added fraction of SiC nanowhisker. The result indicated that with increasing fraction of SiC nanowhisker into the samples, the flexural strength gradually decreased. When the added fraction of SiC nanowhisker was 3.75 wt.%, the flexural strength of the obtained cemented carbides was only about 1000 MPa, which was approximately 1000 MPa lower than that of the sample without SiC nanowhisker. The reason for such phenomenon can be explained as follows. First, the bending test was sensitive to defects, such as micropores and agglomeration in the samples. As mentioned in Section 3.2, the addition of SiC nanowhisker would decrease the wetting ability of binder phase and suppress the migration of liquid binder phase, which was harmful to the densification of the samples and caused more defects in them. These defects may initiate the crack during the bending test. With more SiC nanowhisker, its agglomeration became more serious as shown in Figures 3 and 4, which would be also harmful to the flexural strength. Moreover, the flexural strengths of the produced Ni_2_Si and graphite by the reaction between Ni and SiC nanowhisker were relatively lower than those of Ni and SiC whisker, which would also reduce the flexural strength of the obtained samples. In addition, the reaction consumes the Ni binder, which was confirmed by the decreased amount of survival Ni binder and deep dimples as seen in Figure 3 with increasing fraction of SiC nanowhisker into the sample and would weaken the combination between WC grains. Consequently, the flexural strength of the samples decreased with increasing fraction of SiC nanowhisker. Such results suggested that, because of the reaction between Ni and SiC nanowhisker during SPS, the addition of SiC nanowhisker was of no much help to improve the flexural strength of WC-Ni cemented carbides during SPS.

## 4. Conclusions

Ultrafine WC-Ni cemented carbides with varied fractions of SiC nanowhisker were fabricated by SPS at 1350°C with the assistance of VC and TaC as WC grain growth inhibitors. The reaction between Ni and SiC nanowhisker happened during SPS, forming Ni_2_Si and graphite, which was different from the results of WC-Ni cemented carbides fabricated by hot-press sintering. With the addition of SiC nanowhisker, the average WC grain size decreased from 400 to 350 nm. However, with increasing fraction of SiC nanowhisker, more and more Si-rich aggregates appeared. The Vickers hardness initially increased and then decreased with increasing SiC nanowhisker fraction, reaching its maximum value at about 24.9 GPa when 0.75 wt.% SiC nanowhisker was added. With increasing fraction of SiC nanowhisker, the flexural strength gradually decreased.

## Figures and Tables

**Table 1:** Specifications of the raw materials applied in this work.

	WC	Ni	VC	TaC	SiC nanowhisker
Purity (wt.%)	>99.5%	>99.9%	>99.0%	>99.0%	>99.0%
Oxygen content (wt.%)	<0.31%	—	—	—	<0.5%
Size	0~1 *μ*m (97.5%) 1~2 *μ*m (2.5%)	0.7 *μ*m	2–4 *μ*m	1-1.5 *μ*m	Diameter ≤ 250 nm; aspect ratio ≥ 20

**Table 2:** Nominal compositions of the designed samples in this work.

Sample no.	Sp-0	Sp-1	Sp-2	Sp-3	Sp-4	Sp-5
WC (wt.%)	91.00	90.25	89.50	88.75	88.00	87.25
Ni (wt.%)	8.0	8.0	8.0	8.0	8.0	8.0
VC (wt.%)	0.7	0.7	0.7	0.7	0.7	0.7
TaC (wt.%)	0.3	0.3	0.3	0.3	0.3	0.3
SiC nanowhisker (wt.%)	0	0.75	1.50	2.25	3.00	3.75

**Table 3:** The constituents of the black aggregates in the prepared WC-Ni cemented carbide with 3.75 wt.% SiC nanowhisker as shown in Figure 3(f).

Elements	Spectrum 1	Spectrum 2
C (at.%)	10.3	8.6
O (at.%)	68.6	70.1
Si (at.%)	21.1	21.3
